# “I should have started earlier, but I was not feeling ill!” Perceptions of Kalenjin women on antenatal care and its implications on initial access and differentials in patterns of antenatal care utilization in rural Uasin Gishu County Kenya

**DOI:** 10.1371/journal.pone.0202895

**Published:** 2018-10-03

**Authors:** Roselyter Monchari Riang’a, Anne Kisaka Nangulu, Jacqueline E. W. Broerse

**Affiliations:** 1 Department of Sociology and Psychology, School of Arts and Social Sciences, Moi University, Eldoret, Kenya; 2 Athena Institute, Faculty of Science, Vrije Universiteit Amsterdam, The Netherlands; 3 Commission for University Education, Nairobi, Kenya; 4 Department of History, School of Arts and Social Sciences, Moi University, Eldoret, Kenya; University of Michigan Medical School, UNITED STATES

## Abstract

**Aim:**

The aim of this study was to explore how Kalenjin women in rural Uasing Gishu County in Kenya perceive antenatal care and how their perceptions impede or motivate earlier access and continuous use of antenatal care services.

**Methods:**

A study was conducted among 188 pregnant and post-natal mothers seeking care in 23 rural public health facilities. Gestational age at the initial antenatal care booking was established from their medical cards. Further researcher-administered questionnaire with closed and open-ended questions was used. Key informant interviews with traditional birth attendants (n = 6) and maternal and child health nursing officers (n = 6) were also conducted for triangulation. Descriptive statistics were applied using SPSS programme. The interviews of women who gave consent to be audio recorded (n = 52) were transcribed and thematically analysed using MAXQDA program, based on Andersen and Newman’s (1973) behavioural model of health services utilization.

**Results:**

The mean gestational age at booking initial biomedical care was 23.36 weeks. Only 18 patients (10%) booked before 13 weeks and 45% made four or more visits. The main reasons given for early booking were: illness in index pregnancy (42%) checking the foetus position and monitoring foetus progress (7%). The main reasons given for late booking were: no reason (31%), was not feeling sick (16%), fear or shame due to unexpected pregnancy (13%). Almost half of the respondents (44%) used both biomedical and traditional antenatal care services. Main reasons for visiting traditional care were to: check foetus position and reposition it (63%), collect medicinal herbs (31%), relief discomforts through massage (18%).

**Conclusion:**

Early antenatal care booking is meant for women with unpleasant physical signs and symptoms. Later ANC is meant to check foetus position and reposition it to cephalic presentation and monitor its progress and this is only possible if the foetus is large.

## Introduction

Antenatal care (ANC) is regarded as a key entry point for pregnant women to receive a wide range of essential health-promotion and disease-prevention services. It is considered an effective means for reducing the risk of maternal morbidity and mortality, especially where the general health status of women is poor [1]. Late timing of the first ANC booking is associated with adverse maternal outcomes, because it limits the amount and quality of care that a pregnant woman receives and delays identification and mitigation of risk factors in pregnancy [2]. Studies have shown that women who book ANC late more often suffer from medical complications such as anaemia, hypertension, diabetes and intrauterine foetal death[3]. It is also associated with pre-term and complicated delivery due to undetectable HIV/AIDS [4]. Underweight births and stillbirths are also associated with women who book ANC late [5]. ANC is important in detecting women with a greater risk of delivery complications so that they can be advised to seek medical care. ANC not only avoids complications associated with pregnancy, but also educates women on how to prepare for labour, the post-natal period, family planning and appropriate breastfeeding practices [2]. Studies have further confirmed that women who began ANC early are more likely to opt for skilled professional assistance when they deliver than those who started ANC late [6–8].

The World Health Organization (WHO) therefore recommends pregnant women to begin ANC as soon as a woman becomes pregnant and that low-risk women (healthy women with no underlying medical problems) should have at least four visits at specified intervals (first visit at around 8–12 weeks, the second at 24–26 weeks, the third at 32 weeks and the fourth at 36–38 weeks) for essential interventions to be effectively administered [2].

ANC coverage is a success story across Africa, since over two-thirds of pregnant women (69% in Africa as a whole, and 90% in Kenya) have at least one ANC contact [1]. Studies have found, however, that most women book ANC late (third trimester), whereas others make only one visit or no visits at all [2,9–11], limiting opportunities for quality care and interventions. The tendency to book ANC late is often high among rural communities [3,5,10–12], and the situation is worse in some parts of the country whose health status is generally poor. For instance, in Uasin Gishu County of Kenya (where this study was conducted), only 22% of pregnant women attended at least four Antenatal health clinics (ANC) and only 30% of total deliveries are conducted by skilled health staff [13].

The decision to seek ANC is determined by a mix of cultural, social, economic, geographical and organizational determinants. In the literature, the most commonly cited factors are quality of care, which is mostly operationalized from structural dimensions such as the availability of drugs, staff attitude, waiting hours, staff availability, and services, all of which discourage women from booking ANC early [14–16]. Place of residence (rural or urban) is another factor since rural women are less likely to book ANC early than those in urban areas [6,8,17]. Banda [18], in his comparative study on rural and urban women in Zambia, established that late ANC remains high in both rural and urban areas. Distance to the health facility is another factor and has been shown to have a mixed effect. Some studies suggest that those who live far away from the health centre book ANC late [3,17], others have established that ANC frequency increased with distance from the health facility [5], others indicate that physical accessibility difficulties rather than distance to health service was a factor [8,15] whereas other state that distance itself is not a factor, but the money needed for transport was a barrier [19].

Studies also suggest socio-economic factors specifically education (more educated mothers tend to seek ANC earlier than less educated mothers [5,8,15,16,20]), age of the mother [20,21], ethnicity[6], religious background [12,16,22], household wealth [8,22] and marital status [6]. It is also indicated that women with low parity favoured earlier ANC booking than their counterparts [6,8,21,23]. Further findings indicate that women with complications in the previous pregnancy are also associated with early ANC booking than their counterparts [3,23]. On contrary to these findings, Onoh et al.’s [24] study in South-east Nigeria established that socio-demographic characteristics such as parity, level of education and family income do not influence the gestational age at ANC booking. Onoh et al [24] further disapproved complications or illness in previous pregnancy and instead they established that illness in the index pregnancy and personal wish were significant determinants to ANC booking. Illness in index pregnancy as determining factor for booking or not booking ANC early was also established in other studies [18,19,25–27]. These mixed reactions clearly indicate that the decision to book ANC is a complex decision that is influenced by more than just structural, geographical and socio-demographic variables which are dominating especially in the Kenyan literature [5,8,14,16,17]. Socio-cultural influences provide useful information but not all studies collect detailed information at the personal level thus there is need for more contextualized research. On the other hand these studies mainly focus on the utility of general maternal healthcare services such as delivery and frequency of ANC services while others [6,8,17] rely on information from secondary data (e.g. the Kenya Demographic Health Survey (KDHS) to compute their findings.

Pregnancy is a cultural phenomenon because it takes place within a cultural context. It is perceived differently depending on a population’s social-cultural set-up and their everyday life experiences, and this undoubtedly can influence women’s response to, and use of, ANC services–hence the need for a contextualized research study. Insight into drivers of seeking initial ANC services may contribute to the design of more efficient and cost-effective ANC systems. This study therefore specifically investigates the trend on gestational age at which women commence ANC (both with a TBA and a health facility), their perceptions of ANC and how these perceptions influence differences in commencement and continued use of ANC services.

## Methods

### Study setting and sampling procedures

Data for this study were collected as part of broader research investigating the social cultural context of nutrition in pregnancy and the utilization of nutrition intervention services in rural Uasin Gishu County in western part of Kenya. Maternal nutrition interventions in Kenya are offered free of charge in all government hospitals as part of routine ANC services [28]. Women’s utilization of ANC at health facilities plays a crucial role in uptake of these interventions. Exploring factors that influence ANC attendance was therefore a key objective of this research. For this reason, women who had at least one prior ANC visit in a health facility during the current pregnancy or had delivered a baby within one month were recruited for the study in order to elicit their experiences with nutrition interventions during their previous appointments.

Uasin Gishu is one of the 47 counties of Kenya and it covers a total area of 3,345.2 km^2^ with a total estimated population of 1,023,656 [13]. Most settlements are rural (64.1%). The climatic conditions and soil type in this region are generally favourable for a wide range of livestock and crop production with an average rural land holding of 5 ha, hence the County is commonly known as the “country’s food basket” [29]. However, despite the food surplus in the county, maternal malnutrition indicators of Uasin Gishu are worse than the national norm, particularly with respect to stunting; statistics indicate 31.2% of children in Uasin Gishu are stunted compared to 26.0% nationally [8]. To establish the social cultural context of maternal nutrition in the county, Uasin Gishu County was thus purposively selected. The Kalenjin are the predominant ethnic population in Uasin Gishu County. This ethnic group is composed of smaller sub-ethnic groups (the Kipsigis, Nandi, Tugen, Keiyo, Marakwet, Pokot, Sabaot and the Terik) that share a common dialect and similar cultural traits. The Nandi occupies the largest settlement in Uasin Gishu County, followed by the Keiyo.

There are 171 health facilities in the county, of which 90 are government owned and offer maternal care services free of charge [13]. Most of the facilities are concentrated in the county headquarters (Eldoret town). Uasin Gishu county is administratively divided into six sub-counties namely: Turbo, Soy, Moiben, Kapseret, Kesses and Burned forest. Each sub-county has a sub-county hospital equipped with one medical doctor, nurses, clinicians, a delivery room and maternity wards. However, these sub-county hospitals do not provide maternal services for high-risk women and so refer such cases to the county hospital, which is only one. These sub-county hospitals are the largest facilities in the rural areas and thus serve as referral centres within each sub-county. There are also other health centres (headed by a clinical officer) and dispensaries (headed by nursing officer) which offer ANC services for normal pregnancies but they are not equipped to attend deliveries.

Study subjects were selected from the six sub-county hospitals. Pregnant women attending ANC between March and June 2017 were enrolled. Only Kalenjin women, who had at least one prior visit to an ANC during the current pregnancy or post-natal care within one month, were included. The number of women seeking care in the previous 6 months was determined by reviewing maternal-care registration records. This was used to estimate the number of women who would be attending the clinic during the period when the study was to be implemented. As per the hospital records, approximately 60–240 women seek maternal care per month in each of the six sub-county hospitals. Thus on average, a total 795 women were seen per month in these hospitals. Systematic sampling technique was used to select study participants where by every second woman who met the inclusion criteria was recruited until the minimum desired sample size of 188 was attained. This selection criterion excluded the following women: non-Kalenjin, pregnant and visiting ANC for the first time, unable or unwilling to participate.

### Data collection

A researcher-administered questionnaire with closed and open-ended questions (S1 Doc) was chosen in order to provide room for probing, clarity of questions and enable participants to express their views on the topic in order to generate rich detailed insight information [30]. The questionnaire was developed after a literature review and discussions with nursing officers in charge of Maternal and Child Health (MCH). The topics covered in the questionnaire included: demographics, reasons for early ANC booking, reasons for late ANC booking, whether the interviewee had ever used TBA services for the current pregnancy, the gestational age and the number of times they visited TBA care, the nature of TBA care and their opinions on both TBA and ANC services.

The data-collection exercise was coordinated and conducted by the first author with the help of four research assistants who were fluent in both local Kalenjin dialects, English and Swahili languages, with social science research experience. The research assistants were properly trained in the research instruments, language translation and they participated in the pilot study and review of research instruments after piloting to ensure consistency and inter-researcher reliability. Data were collected in the local language or Swahili depending on the preference of the respondent. If the respondent consented, her responses were noted and recorded and later transcribed verbatim and translated into English for analysis. If a respondent objected to being recorded, detailed notes were taken. Individual face-to-face interviews were preferred because they give the opportunity to observe respondents’ facial expressions and body language, particularly important for correct interpretation of the answers [31]. The individual interviews were conducted in a quiet private room at the health facilities to avoid distractions, ensure privacy and anonymity of the responses and enhance crystal-clear recordings [32]. Each woman was interviewed once and the interviews lasted for 30–45 minutes.

The reliability of the findings was ensured through triangulation using cross-checking questions, observation, key informant interviews with TBAs who were also herbalists (n = 6) and nurses offering ANC (n = 6). The health workers were interviewed at their place of work whereas TBAs/herbalists were interviewed either in their homes or at the market centres where they sell their herbal medicines. The TBAs were identified by snowball and convenience sampling through respondents who gave birth at home and who took herbal remedies during pregnancy. Information gathered from key informants was further employed to explore meanings and enrich the responses obtained from the interviews with pregnant women.

### Ethical considerations

Research approval was obtained from the National Commission for Science, Technology and Innovation (NACOSTI/P/15/2335/5353; 2-Apr-2015) (S2 Doc). NACOSTI is a state corporation with the overall mandate to review and regulate the quality of science in the country and approve research studies. NACOSTI offers the researcher approval to conduct research activities in the community only if the study does not require further clearance from an ethical institutional review board. In this case, the study was not recommended for further ethical review. For this study additional permission were obtained from the Uasin-Gishu County Director of Health, the County Commissioner and the County Director of Education (S2 Doc). Institutional approval was obtained from the Moi University (Kenya) and the Vrije Universiteit Amsterdam (Netherlands) (S2 Doc), to undertake this research. Study participants were provided with information about the study before any consent to participate was sought. The participants were also informed of their right to abstain from participating in the study, or to withdraw from it at any time, without reprisal if they felt uncomfortable to continue with the study. Measures to ensure confidentiality of information was also provided. All respondents provided written informed consent to participate in the study. Informed consent from adolescents below 18 years was guided by Fisher et al’s (2003) recommendation that unlike younger adolescents, those over 16 can make informed decisions as well as adults [33]. Ruiz-Canela et al 2013, further recommends that “If adolescents are mature enough to understand the purpose of the proposed study and the involvement requested, then they are mature enough to consent” [34]. However in this case, the consent forms were read out to the adolescents in the presence of a legal guardian/parent and informed assent was sought from minors, while legal guardians/parents gave written informed consent.

### Data analysis and theoretical framework

Statistical data were coded and analysed using SPSS software (version 23) to establish frequencies of descriptive statistics and the results were tabulated. The recordings were transcribed verbatim and translated into English with each participant being identified with a pseudonym, and these are used as narratives in the results section. With the help of MAXQDA 12.3.2 software, both notes and voices were further coded into themes and sub-themes based on Andersen and Newman’s behavioural model of health services utilization [35] as the initial coding guide.

The Andersen and Newman model used in this study specifically focuses on individual determinants of general patterns in the use of health services. The underlying model assumes that use of health services is dependent on: (1) the predisposition of the individual to use services; (2) enabling factors (individual’s ability to secure services); and (3) need for care (individual’s illness level).

Predisposing factors refer to the propensity to use health services and this can be predicted by individual characteristics that pre-date the onset of specific episodes of illness. These factors include demographics (e.g. age, sex, marital status and past illness), *social-structural* characteristics (e.g. education, occupation, family size, ethnicity, religion and residential mobility) and attitudinal-beliefs (e.g. values concerning health and illness, attitudes towards health services, and knowledge about medical care, physicians, and disease) of individuals, which influence people’s attitudes towards illness and care. According to Andersen and Newman predisposing variables as such are not considered to be a direct reason for using health services but do result in differences in inclination towards using them.

Enabling factors: Enabling variables refer to means that make health service resources available to the individual patient and may emanate from the family or community. Enabling factors include family resources, such as income, level of health insurance coverage, or other source of third-party payment, whether the individual has a regular source of care, the nature of that regular source of care, and the accessibility of the source. In addition, the community resources in the area in which the family lives can affect the use of services, e.g. the number of health facilities and personnel in a community, the price of health services, region of the country and the rural or urban nature of the community. These variables might be linked to use because of local norms concerning how medicine should be practised or overriding community values that influence the behaviour of an individual living in the community.

Illness level: Individuals or their family must perceive illness or the likelihood of its occurrence for them to make use of health services. Illness level represents the most immediate cause of using health services. Measures of perceived illness include number of disability days (days during which individuals are unable to do what they usually do, be that work, go to school, take care of the house, or play with other children), symptoms experienced in a given time period, and a self-report of general state of health (e.g. excellent, good, fair, or poor). In addition, evaluated illness measures determine the need for care. These measures are attempts to get at the actual illness that the patient is experiencing and the clinically judged severity of that illness. The symptoms reported by the individuals can also be weighed by physicians regarding the probability of need for care.

**Fig 1:**
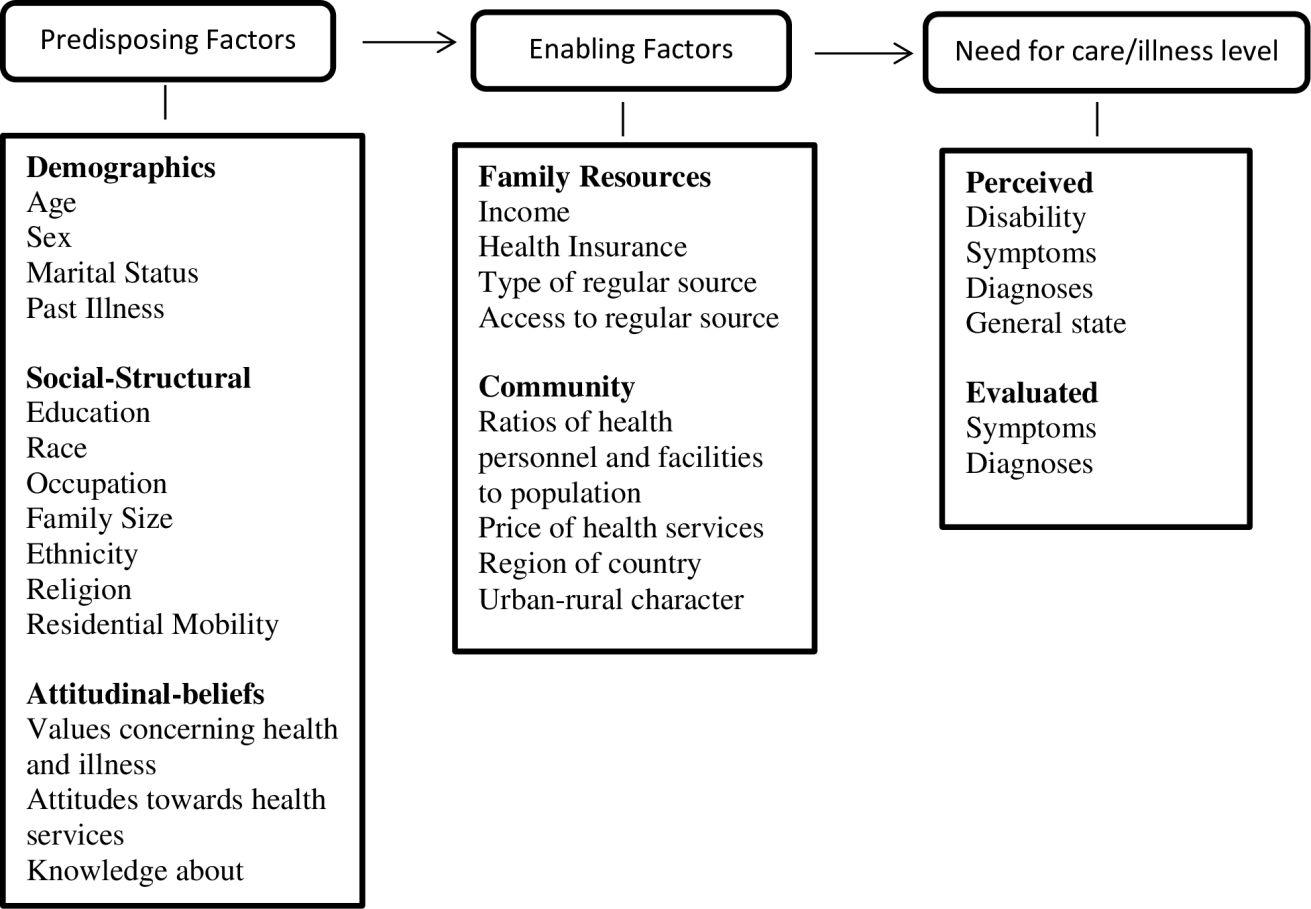
Individual determinants of health service utilization (Andersen and Newman, 1973:14).

According to this model, therefore, patients must perceive a need for care, they have to respond to this need and the patient’s environment must enable the search for care. Fig 1 presents each of these components of the model and the variables that operationalize them as established by Andersen and Newman.

**Fig 2:**
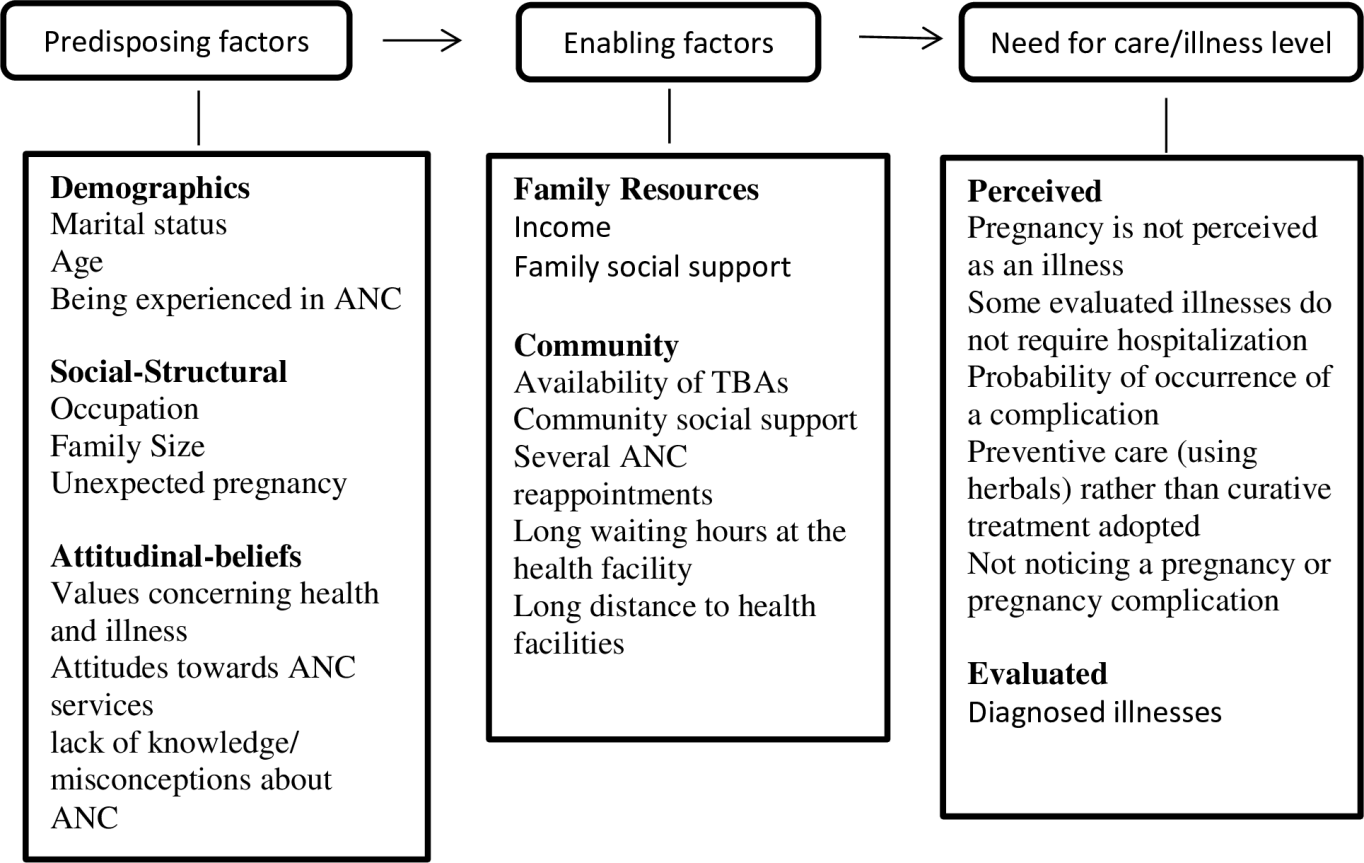
A model on factors influencing differentials in ANC access in Uasin Gishu County, Kenya.

This model thus actively facilitated the exploration of respondents’ perception on ANC, integrating these perceptions into predisposing, enabling and need for care contexts to producing one behavioural outcome regarding maternal ANC seeking behaviour. Newly emerging codes in the transcripts were inductively added to the framework’s variables that correspond to the findings of this study to build our model of personal factors influencing ANC-seeking behaviour as indicated in Fig 2. When new codes or themes were added to the framework, all data were re-scrutinized several times to obtain a sense of the whole. Researchers with different backgrounds provided input to the analysis to increase its validity [36].

## Results

Statistical results of the research findings were first presented in tables. In total, 188 women were interviewed. Their demographic details are presented in Table 1.

**Table 1:** Demographic characteristics of the respondents (n = 188).

Indicator	Characteristics of women	Distribution (N)	Distribution (%)	average
*Sub-county*	Ainabkoi	23	12	
	Kapseret	34	18	
	Kesses	21	11	
	Moiben	48	26	
	Soy	41	22	
	Turbo	21	11	
*Place of Residence*	Rural home	128	68	
	Rural centre	60	32	
*Tribal affiliation*	Nandi	104	55	
	Keiyo	51	27	
	Marakwet	13	7	
	Tugen	13	7	
	Kipsigis	5	3	
	Sabaot	2	1	
*Maternal status*	Pregnant mothers	102	54	
	Recently delivered	86	46	
*Gravida*	Primigravida	72	38	
	Multigravida	116	62	
*Age (years)*	≤19	15	8	
	20–24	79	42	
	25–29	59	31	
	30–34	22	12	
	35–39	10	5	
	≥40	3	2	
	*Mean age*			*25*.*5*
*Marital status*	Never married	28	15	
	Married	160	85	
*Educational level*	Primary	82	44	
	Secondary	75	40	
	Post-secondary	31	16	
*Occupation*	Farmer	74	39	
	Housewife	24	13	
	Casual labourer	6	3	
	Business	44	23	
	Student	27	14	
	Salaried	13	7	

As indicated in Table 1, a total of 188 women were interviewed and these were either pregnant (102) or had given birth in the past month (86). The majority of the women spoke Nandi (55%) and Keiyo (27%) dialects, because these are the dominating sub-groups of the Kalenjin in Uasin Gishu County[29]. The respondents were equitably distributed across the six sub-counties of Uasin Gishu, with Moiben and Soy commanding a larger share (26% and 22% respectively). The mean age was 25.5 years ranging between 16 and 45 years of age, and most (85%) reported being married. In terms of education, 44% and 40% of the women had either completed or not completed primary and secondary education, respectively. Most of these women (62%) were multigravida (2–9 pregnancies) and more than half were engaged in the informal economy as farmers (39%), housewives (13%) or running small businesses (23%) while 14% were students/pupils.

### Gestational age at commencement of antenatal care services and frequency of access to care

The analysis shows that the distribution of the timing of the first ANC visits range from the first trimester to the ninth month of pregnancy, with a mean of about five months (Table 2)

**Table 2:** Information on commencement and frequency of ANC services.

Indicator	Characteristics of women	Distribution N (%)	Mean
Type of ANC accessed (n = 188)	Did not access any ANC	2 (1)	
	Accessed health facility care	186 (99)	
	Accessed both health facility and TBA care	82 (44)	
Gestational age at first ANC visit (weeks) N = 186	<13 weeks	18 (10)	
	13–19.9	27 (14)	
	20–26.9	95 (51)	
	≥27	46(25)	
average			*23*.*4 weeks*
Gestational age (months) at first TBA care visit (n = 82)	≤3	20 (24)	
	4–6	34 (42)	
	≥7	28 (34)	
Average			5.3 months
Frequency of health facility based ANC (Number of times) (n = 86), (based on recently delivered mothers)	1	4 (5)	
	2	14 (16)	
	3	30 (35)	
	4	22 (26)	
	≥5	16 (19)	
Frequency of seeking TBA services (number of times) n = 78)	1	34(41)	
	2	24(29)	
	3	10(12)	
	4	5(6)	
	≥5	9(11)	

Table 2 shows that all respondents (except 2) had accessed ANC care services at the health facilities, but only 10% did in the first trimester and 45% achieved the minimum four or more visits recommended in WHO guidelines. On average, these women started their first ANC at a health facility at a gestational age of 23.4 weeks. Slightly more seek TBA care in the first trimester (26%), but their frequency (more than four times) is lower (11.5%). The majority of the respondents did seek TBA services once or twice during their pregnancy period.

### Barriers or motivations to start ANC early

Various reasons as to why pregnant women in the study area book ANC early or late were investigated and these are presented in Table 3.

**Table 3:** Influences on initial commencement to ANC and perceptions of ANC Services.

	Reason	Frequency and (percentages)
Reasons for late gestational age (≥20 weeks) at first ANC (n = 142)	No apparent reason	42(31)
	Was not feeling sick	22(16)
	Fear, and shame (due to unexpected/unwanted pregnancy)	16(13)
	Doctor’s recommendation	9(7)
	Did not know she was pregnant	9(7)
	To reduce the number of trips (ANC attendance is cumbersome / tiresome /tedious)	9(7)
	Too small to be felt	5(4)
	Did not know the right time to commence ANC	5(4)
	Had visited TBA	2(2)
	Already has experience	2(2)
	Was on duty	2(2)
Reasons for early gestational age (<20 weeks) at first ANC booking (n = 45)	Was feeling unwell	19(42)
	To check if the baby is progressing well	7(16)
	Was told by mother to begin ANC	4(9)
	To check mother’s blood (amount of blood and HIV status) and check if the baby is fine	4(9)
	Was advised by doctor	3(7)
	Personal decision (no reason given)	3(7)
	To be able to detect pregnancy complications early	2(4)
	Had experienced previous miscarriages	1(2)
	Advised by employer	1(2)
	To give birth to a healthy baby	1(2)
Reasons for visiting TBA services (n = 82)	To check whether the baby is well positioned; TBA reassures the position of the foetus or repositions the foetus if not in the correct side	52(63)
	To collect medicinal herbs (reported by both the sick and non-sick respondents)	31(38)
	Numb legs and difficult to walk because of too much pressure of the baby on one side; TBA turns the baby, relieves pain and discomfort	15(18)
	Lower back and abdominal pain. TBA gives pain relieve and curative herbs.	6(7)
	To confirm expected date of delivery (EDD).	5(6)
	Pre-term labour/cramps. TBA gives preventive herbs.	4(5)
	Nausea, lack of appetite and vomiting; TBA gives herbs to counter the feelings and increase appetite.	3(4)
	The baby was not making any movements in the uterus; TBA gives herbs massages and repositions the baby to enhance movements.	2(2)
	Vaginal bleeding; TBA gives herbs that stop the bleeding.	1(1)
Opinions on general ANC Services at the health facilities (n = 186)	Not satisfied	11(6)
	Satisfied	175(94)
Opinions on general TBA care Services (n-82)	Not satisfied	1(1)
	Satisfied	81(99)

As indicated in Table 3, most of the respondents (31%) who did not start ANC early did not give any apparent reason for the delay. For the rest of the respondents, it was either they were not feeling ill (19%) or they were scared/ashamed of disclosing their pregnancy because it was unexpected, untimed or unwanted (13%). Other reasons for late booking of ANC that were reported by less than 10% of the respondents included: doctor’s advice to book late, late recognition of pregnancy, whereas others believe that booking late will reduce the monthly ANC appointments, which they find cumbersome, Other respondents did not know the right gestational age to book for initial ANC, whereas other respondents believed that in early pregnancy (before 5 months) the baby will be too small to be felt/examined. Almost half of the women who booked ANC early was because they were ill (42%). Other respondents (16%) wanted to know whether the baby was progressing well, or they were advised by their mother (9%) or the health officer at the hospital (7%), while others (9%) wanted to test their blood or did not mention any reason for booking early (7%). Almost all respondents (99%) were satisfied with TBA services. This was slightly higher than for those seeking care at the health facility (94%).

On the other hand, women could go for multiple services with the TBA. The main reason given by the majority of the women respondents (63%) was to check whether the baby was properly positioned in the uterus. If wrongly positioned, the TBA will turn the foetus to correct the position. This will help lighten the pregnancy, reduce discomfort and numbness of the leg (especially on one leg if the baby is lying to one side), or complicated delivery if the baby is lying horizontally. Other reasons were to seek treatment or herbal care from the TBA (38%). These reasons are the same for those seeking care at the health facility except checking of blood, although the mode of service delivery varies as reported by the respondents in the next section.

### Perceptions of ANC and differentials in ANC utilization patterns

The research findings were categorized into three major themes of individual determinants of differentials in use of ANC services, based on Andersen and Newman’s behavioural model of health care utilization [35], that is: predisposition factors, enabling factors and need for care. These major factors were operationalized into categories and sub-categories based on emerging and part of the predetermined variables of Andersen and Newman’s behavioural model.

### Predisposition factors

#### Demographic

In this study, of the demographics stated by Andersen and Newman, we established age and marital status as influencing variables. Elderly mothers and very young unmarried women (in most cases pupils/students) tended not to book ANC early.

In addition to age, previous knowledge and experiences with ANC were also established to influence the use of ANC in the present pregnancy. Two multigravidae women reported at ANC late because they felt they already knew how to manage a pregnancy based on their previous experiences without necessarily visiting a health facility.

#### Social structural characteristics

In this study, occupation and family size were the only social-cultural characteristics established. Social perceptions of pregnancy make women feel proud or ashamed of being pregnant and this greatly influences their initial ANC booking. Some respondents (16%) registered at ANC late because of being ashamed of their pregnancy mainly because they had conceived unexpectedly. Those who felt ashamed were mostly the students/pupils. They were afraid of negative reactions and of being punished by teachers, peers or parents. One respondent, who attended secondary school, had a pre-term birth and never made any ANC visit:

“I realised I was pregnant while I was still in school. I could really hide my pregnancy by acting like I am not. I could participate in all school activities including very active sports so that I cannot be suspected to be pregnant. It was painful but I could pretend and that is what I think caused my pre-term birth. …….no, if I seeked TBA or health facility care, they might tell my mum and I really did not want anybody to discover this……., even when I was in labour, I secretly took a motorbike to the hospital…., the nurses called to inform my mum after I delivered.” R170404_002

Other respondents did not start ANC because they conceived too close or at a late age or already had many children.

I was not totally expecting a pregnancy; I received the pregnancy test results with a shock and stress. I went home to swallow the shock first (to calm). Only after many months I accepted the pregnancy and decided to begin my ANC clinics. R170404_003

#### Attitudinal-beliefs

In this study, 76% of women who seek care at the health facilities also do consult the TBAs. Many of these women pointed to the different nature of services offered at the health facilities and the pregnant women’s expectations:

“Something has been pressing my leg. One morning I woke up it was worse, I could not walk well and I was feeling very tired……. I came to the clinic and explained to the nurse and she told me it is normal for pregnancy. …. On my way home, I passed by a TBA to ‘hold the tummy for me’ [massage the uterus] and actually the pain reduced and I felt lighter.” P170404_003

“The baby was lying on one side and could not play well. The TBA checked it for me and she repositioned the baby and the baby started playing well…… The nurse just touched my tummy; she did not turn the baby to position.” R170405_001

“These nurses they do not know anything. They just touch the tummy and they don’t tell you anything. That touch is very light; it cannot detect any possibility of foetal malpresentation or multiple births.” R170405_002

Some women doubted medical feedback reports and opted to get confirmation from TBAs after attending the health facility:

“I was having pain in my tummy; I came to the hospital first and they told me it is due to the heavy duties I do. …. I went to the TBA, she held my tummy and told me that the baby has moved down because I do a lot of work. She worked on it (massaged) until it returned back to position. Every time I go there she tells me I should stop doing a lot of work.” R170420_002

“They wrote for me an EDD of 24 April. I suddenly started feeling some strange pain in my tummy before due date. I went to the TBA she touched my tummy, told me the baby is ready to come out. She told me to go home; if it worsens I go to hospital.” R170418_001

Nurses also do not offer certain services which these women opt for:

“She used soap and water to make it smooth, massaged the tummy, one hand holding down another one up, then sideways until the baby move up to position. … By the way she helped me a lot, don’t underrate them.” P170424_001

However, some respondents do not like the TBA’s services while others decline their services having tried and failed to be cured.

“I do not want those women [TBA], another woman explained to me the way it is painful! …. you know the hospital is better because they can tell by a light touch because they are educated on that line.” R170418_005

“I was feeling a lot of pain in my tummy like the baby is ready to come out. I went there [TBA] twice, she gave me herbs but the pain did not stop. So I never went back to her again.” R170410_005

Lack of knowledge of or misconceptions about ANC was also established in a number of respondents as a reason why they booked their initial appointments late. As indicated in Table 3, the majority of the respondents (31%) who did not start ANC early did not have any apparent reason for the delay. This is an indication that they do not clearly understand the bio-medical meaning of ANC. Most of the responses given in this case were:

“No reason…I just stayed.**”** M170502_001

“I did not have any reason, I just kept on saying I will go but when the day comes I don’t go, until I realized I am clocking to seven months.” R170405_003

Other respondents booked late because they do not know the correct gestational age to commence ANC.

“I normally start ANC for my pregnancies at a gestational age of 24 weeks. And that is why also this pregnancy I waited for 24 weeks.” S170418_002

Some respondents reported that it is recommended for a pregnant woman to make a minimum of four ANC visits. In their calculation, they concluded that 5–6 months is the ideal gestational age for booking ANC to be able to accomplish the minimum requirement by the end of their pregnancy. This reasoning can validly confirm why the majority of the Kalenjin women (51%) mostly book their first ANC between the gestational ages of 20 and 26 weeks.

“I did not know people commence ANC care early; I know people start once it gets to six months. And that is why I waited for six months… so that one can make at least the recommended four ANC visits.” 170424_002

This misconception seems to be created and reinforced by some of the clinicians at the health facilities, as confirmed by 7% of the respondents from different health facilities:

“I started my ANC at six months because it is advisable to attend ANC at least four times…. I learned this during my first pregnancy because during that time, I came early and I was not given pregnancy recommended drugs (supplements) because the pregnancy was too young to be given.” [16weeks]. R170410_011

“I went to book for my ANC at XY clinic when I was three months pregnant, they told me I should start ANC at a gestational age of five and a half or six months. I went home and waited for the six months. And that is how I came to know that one should start ANC after six months.” P170404_003

Some practitioners do advise patients to begin their ANC early, as reported by other respondents.

“During my second month, my medical tests confirmed I was pregnant. The doctor told me to begin my ANC clinic appointment immediately, and that is why I started early.” *R170420_003*

However, “booking early” is not clearly comprehended by some respondents.

“During my previous clinic, we were told ‘you should begin your ANC immediately you notice you are pregnant’ therefore I decided to start my ANC for this pregnancy early [20 weeks].” R170406_002

The same misconception of beginning ANC at six months is being passed on by the elderly women to the next generation of new mothers:

“I was told at home, clinic begins at the 6^th^ month. I did not know that it should begin immediately….my mother-in-law told me.” 170411_004

However, some other respondents believed that ANC should begin when the pregnancy is “big”:

“…what! Two months! Now what is that? It is too small, you cannot even hold it. How can that pregnancy be examined?! That is being too excited of a pregnancy…people might even laugh at you.” R170418_002

### Enabling factors

#### Family resources

In this study, only two respondents reported ANC late because of lack of money for laboratory tests, probably because free maternal care policy was introduced by the Kenyan government in 2013 [37].

“I did not know they increased laboratory test charges to Ksh. 350. I thought they still charge Ksh. 100 for laboratory tests just like during my previous pregnancy in the year 2013. So I had to go back home look for more money to top up before resuming the ANC.” R170420_002

Instead, family enabling variables relating to social rather than financial resources were commonly established in this study. Some respondents began ANC appointments because they were encouraged by their mothers or other family members to seek for care.

#### Community resources

In this study, the community enabling factors established were: availability and accessibility of TBA services in the community, social support provided by members of the community and inconveniences experienced at the health facilities.

Many respondents (76%) said they had used TBA care services and two respondents booked initial ANC late because they were receiving TBA services. This is a clear indication that TBAs are easily accessible in the community and that they compete with the health facilities services, thus, affecting the initial booking and use of ANC in the health facilities.

Nine respondents found ANC appointments cumbersome because of the frequent monthly re-revisit appointments they are given. As a result, they deliberately delayed initial ANC booking in order to reduce the subsequent number of ANC appointments.

“…it will be tiring; I will be attending every month until I give birth. I thought if I start at six months I will be given fewer return visits. But if I had a complication I should have started early.” S170502_001

“I have been coming early for all these other five pregnancies, when I conceived this sixth one, I decided to wait until I am seven months.” S170418_001

These feelings might have been exacerbated by long waiting hours at the ANC and distance from the health facilities.

“…those will become very many trips. You know the centre is far!” R170412_003

“I came, I found a long queue, and I decided to go home, that is why I delayed to book ANC.” P170410_001

Other community enabling factors established in this study relate to social support provided by members of their community. Two student respondents from the same school reported that they started ANC late because they did not want it to be known by the school administration. However, when the school administration discovered, they instructed the school matron to drop them for ANC using the school van until they finished their final examinations.

#### Illness level/ need for care

Many respondents did not perceive pregnancy as a condition that requires medical attention; only when some unusual signs and symptoms are experienced is treatment warranted. As indicated in Table 3, 19% of the respondents who did not book ANC early mentioned that they did not have any health problem in relation to their current pregnancy that required medical attention.

“…….I was not feeling any problem.” S170418_004

“I should have started ANC early, but I was not feeling ill! I was just okay throughout the cycle.” M170405_003

The majority of the respondents (51%) who booked their first ANC early did so because they were feeling ill. Various symptoms evaluating their illness level were reported.

“I was having lower abdominal pain, headache, fever, I decided to come [hospital] for examination.” S170420_002

“I was having dizziness, I decided to come for them to measure whether the amount of my blood is fine.” S170502_001

“I was not having appetite and very selective on food, I could vomit everything I ate, I decided to come they give me medication that can give me appetite.” S170502_002

The likelihood of illness was also established as an influencing factor for early booking to ANC health services. One woman booked ANC early because she had experienced successive miscarriages at the third month of her previous pregnancies. Two other respondents booked early because they felt it is important in determining and dealing with possible complications.

However, according to many respondents not all evaluated illness requires hospital care. Symptoms such as numbness of one leg, no movement of the foetus, pre-term cramps and nausea were thought to require TBA tummy massage or medicinal herbs.

“The baby was not playing. I wanted her to hold for me the tummy. She made it and the baby started moving. I could even feel it is well.” R170405_001

“Sometimes mostly after seven months you feel the baby is too heavy! Making movements difficult, you go there, she [TBA] holds it for you, you feel lighter.” R170405_002

“I went to see her (TBA) at 3 months because I was bleeding. The baby wanted to come out. She gave me medicinal herbs, it stopped and the pregnancy started growing again.” R170418_008

Some respondents opt for preventive herbal care, using herbs as immunity boosters against contracting diseases and to prevent mother-to-child disease transmission.

Some respondents (7%) who were not alert to their symptoms did not recognize their pregnancy in good time and delayed their initial ANC booking.

“I did not know I was pregnant because I usually have irregular menstrual periods.” S170418_006

Evaluated illness was also established in this study. Some women started ANC early when their illness was clinically determined following physical and medical examinations.

“I did not know I was pregnant. I came for medical check-up because I was feeling dizzy and unwell. They tested me and realised I was pregnant and my amount of blood was low. They gave me drugs that increase amount of blood and advised me to start ANC appointments immediately.” R170420_003

## Discussion

This study aimed to assess the gestational age at which women in rural Uasin Gishu County Kenya commence ANC both at the TBA and the health facility, their perceptions of ANC and how these perceptions influence the gestational age at commencement and differential patterns in use of ANC services. The results indicate that a large percentage of these women (90.4%) do not seek ANC during their first trimester as WHO recommends. This percentage is higher than the 80.2% of Kenya overall [10], 86% found in rural western Kenya [14], 71% in Tanzania [23], 83.1% and 82.6% in Nigeria [20,24]. Only 45% made the four or more ANC visits recommended by WHO during their pregnancy, which is lower than 58% of Kenya overall [10]. The mean gestational age at booking of 5.8 months is slightly higher than the mean gestational age of 5.1 months in Tanzania [26] and 5.4 months in Kenya in general [10].

### Perceptions of ANC and their implications for commencement of ANC services

The results of this study show that nine out of ten Kalenjin women do not seek ANC during their first trimester and that only 45% made the four or more ANC visits recommended by WHO during their pregnancy. This could be attributed to the fact that, as indicated in the results, the Kalenjin women have their own meanings regarding ANC based on their pregnancy and childbirth experiences, which differ from the expectations and perceptions of modern medicine. This clash between cultural and bio-medical perceptions of ANC explains the trend of ANC behaviours expressed by the Kalenjin women in this study.

One major reason identified in this study to book ANC late is that pregnancy among the Kalenjin community is perceived to be a normal condition that in the absence of certain complications does not necessitate medical attention. Furthermore, some symptoms and complications do not require formal medical care; there are those that require TBA attention or herbal treatment. This is contrary to modern medicine where pregnancy is seen as being associated with high health risks, requiring medical monitoring, and where lack of access to ANC is seen as a deviation from clinical norms or abnormality. The perception of pregnancy as a normal condition that does not require medical attention is common in African countries [19,24,38], and has been established to be a major barrier to seeking formal maternal health care. The tendency not to commence ANC early because of not feeling or being unwell was also established in Nigeria [24,25] and Uganda [19].

On the other hand almost half (42%) of the women who booked ANC early were experiencing abnormalities/complications with their pregnancies. this is a clear indication that as per the Kalenjin community, health facilities are attributed to be centres for the sick or for people experiencing abnormal signs and symptoms. This is contrary to medical guidelines where all pregnant women are expected to attend ANC within the first trimester irrespective of their symptoms.

Similarly, the reason for women to visit ANC while they had no abnormalities usually was that they wanted to know the status of the baby and to have their blood tested in order to know their HIV status or iron level. This is an indication that health facilities are perceived to be a centre for diagnosis by using instruments and laboratory examinations which cannot be accessed via ethno medical care. However, after the diagnosis, studies have indicated that these women have their own local ways of treating various medical conditions such as anaemia among others, using herbal and nutritional remedies[39]. The findings in this study are contrary to the findings among the urban poor in Kenya [40], where women avoid hospital-based childbirth because they would be forced to undergo HIV testing in the hospital and they will be stressed about the reality should they test positive.

In addition, it was also established in this study that these women sense pregnancy abnormalities based on their own subjective physical experiences and sensations such as numbness of one limb, bleeding, loss of appetite, vomiting, or unpleasant physical symptoms such as abdominal pain, lower back pain, and pre-term cramps among others. Each of these symptoms determines the appropriate care provider to address them, who is not always a clinician. There are those that instead require a TBA in order to obtain herbal medicine and massage. According to Helman [41], this is a risky assumption because some dangerous health conditions such as anaemia and high blood pressure, which are common among pregnant women, are asymptomatic and might not be detected easily.

As indicated in the research findings, the majority (52%) of the respondents with no abnormality and who use TBA services mainly wanted to confirm the foetal presentation in the uterus. Breach or traverse presentation is of great concern to these women because it is believed to lead to a complicated delivery. However, the nature of confirmation by the nurses at the ANC clinic is doubted by these women. They trust the TBA more, because TBAs not only assess foetal presentation but also manipulate the baby into the head-down presentation, by external version, a service which is not provided by nurses at the ANC appointments. This indicates that there may be a difference in how these women and obstetricians assess the quality of ANC and how they measure a successful ANC outcome and this is highly likely to be a disincentive to seek ANC at the health facilities. Lack of appropriate knowledge on ANC examination could be triggered by the fact that educational level of almost half (44%) of the respondents in this study is primary education and most of them wok in domestic sector as either housewives or subsistence farmers.

Treating pregnant women as passive, dependent patients was also established as a disincentive to seek ANC at the health facilities. Women reported that some nurses do not give any feedback information regarding their medical examination, especially on foetal presentation, which is of great concern to these women even to the point when they are convinced that the foetus is in a traverse presentation. Based on these women’s great concern on foetal presentation, they cannot see the sense of booking ANC within the first trimester because the foetus is too small to physically detect the position.

Another reason is that five respondents did not know the right time to book for ANC, and this confusion is in some cases caused by incorrect advice given by health care providers. This could be attributed to the fact that the majority of the MCH nurses are members of the Kalenjin community. Hence they are highly likely to bring with them their own cultural ideas, assumptions or prejudices into health practice. A study by Onoh [24] in Nigeria confirms this finding. In his study, he established that counselling on early ANC booking did not affect the gestational age of booking initial ANC, making him doubt the accuracy of messages provided at these counselling sessions.

It is also noted that some women (13%) delay starting ANC because they fear or feel ashamed to disclose their pregnancy when they conceive unexpectedly. Pregnancy among the Kalenjin community is conceptualized as a social stigma and a serious crime especially if it does not fit into “official” versions of reality. A study by Kanogo [42] among the Kipsigis (a Kalenjin sub-ethnic group) established that conception and motherhood before female initiation was unacceptable and condemned because it was considered to be ritually unclean and would pollute the ethnicity. Thus children born in such situations were suffocated to death with mud during birth. Such condemnation seems to have trickled down to the current generation making them fear disclosing their pregnancy status. Most of the women who reported this reason are young (i.e. students and pupils), those with many children, old mothers and those with short interval pregnancies. unfortunately, these are women considered to have elevated risks of pregnancy and delivery because they are highly likely to have pre-term babies and are associated with greater risks of maternal mortality [43,44]. Delayed ANC booking due to shame about disclosing one’s pregnancy is common in Kenya [17,21] and was also established among the married adolescent girls in Bangladesh [36].

Many women reported using herbal medicine during pregnancy, whether or not they were experiencing pregnancy complications. Some of these medicinal herbs, as established in our previous study [39,45], are believed to be immunity boosters and to cleanse the foetus, hence they are recommended to all pregnant women irrespective of their health status. Use of medicinal herbs during pregnancy is a common practice in Kenya [45–48] and more studies on these medicinal herbs could help to better understand their effectiveness or the dangers they may pose. Over-reliance on medicinal herbs could be attributed to infrastructural challenges facing rural communities in Kenya that constrain access to bio-medical health facilities [49–51].

Other nine respondents delay booking ANC in order to reduce the number of trips because they find them cumbersome. A similar finding was established by Kisuule and colleagues [19].

## Limitations of the study

In this study, we applied Andersen and Newman’s behavioural model of health services utilization. This model is more general about using health services and is not specific for perceptions on ANC booking and uptake. We nevertheless still found it applicable in this study although we were not exclusively restricted to the variables of the model, which is why we also adopted open questions and open coding.

## Conclusions

The aim of this study was to investigate the perceptions of Kalenjin women regarding ANC and how these perceptions influence the gestational age at which they commence ANC attendance and differentials in inclination towards using these services. The findings indicate that 90.4% of Kalenjin women do not seek ANC during their first trimester and that only 45% made four or more ANC visits during their pregnancy as recommended by WHO. Most women commence ANC between 20 and 27 weeks or later. According to these women, early ANC booking is meant for women experiencing unpleasant physical signs and symptoms during pregnancy and that each of these symptoms determines the appropriate care provider, who is not necessarily a clinician but may be a TBA. On the other hand, later ANC is perceived as meant to examine the progress of the foetus, assess foetal presentation in the uterus and reposition the foetus. The foetal position cannot be detected through external examination in early pregnancy, and foetal examination without correction–which is common at the health facility–is a great disincentive, and explains what attracts many women to seek TBA care. Other factors that delay initial ANC booking include: misconception on the right time to begin care, unexpected pregnancy, late recognition of pregnancy, and a feeling that early booking will increase the number of monthly ANC follow-up appointments which they find to be cumbersome.

## Recommendations

Based on the findings of this study we recommend the following:

- There is a need for more attention to the education of pregnant and potential mothers, to provide correct messages on the meaning and importance of ANC and the appropriate gestational age to begin care.
- Flexibility in ANC follow-up appointments by MCH nurses should be encouraged. It is important for health workers to categorise pregnant women into low- and high-risk groups, and plan ANC differently for them. This will reduce the number of ANC visits for women with a low-risk pregnancy that tend to reduce delays in initial booking.
- School administrators, parents and guardians should give moral support and encourage pregnant students/pupils to begin ANC early.
- MCH nurses should always listen and share medical examination findings with their patients to ease tension and reduce unnecessary doubts regarding their services and competence.
- Potential mothers and pregnant women should be educated about family-planning methods and encouraged to use them in order to prevent unexpected pregnancies.

## Consent for publication

The people interviewed were informed about the study’s objectives and the eventual publication of the information gathered, and they were assured that the informants’ identities would remain undisclosed.

## Supporting information

S1 Doc Questionnaire-administered. (DOCX)

S2 Doc Ethical clearence. (PDF)

## Abbreviations

ANC: Antenatal Care
MAXQDA: Max’s Qualitative Data Analysis software
HIV: Human Immunodeficiency Virus
RNA: Ribonucleic Acid
HIV RNA: concentrations (viral load levels) in HIV-positive individuals
WHO: World Health Organization
KDHS: Kenya Demographic Health Survey
IFAS: Iron and Folic Acid Supplements
MCH: Maternal and Child Healthcare
TBAs: Traditional Birth Attendants
NACOSTI: National Commission for Science, Technology and Innovation
EDD: Expected Date of Delivery
HB: Haemoglobin
ASALI: A Sustainable Approach to Livelihood Improvement
CIS: Centre for International Studies
SEKU: South Eastern Kenya University
VU: Vrije Universiteit Amsterdam
