# Supplementary material for: “I should have started earlier, but I was not feeling ill!” Perceptions of Kalenjin women on antenatal care and its implications on initial access and differentials in patterns of antenatal care utilization in rural Uasin Gishu County Kenya
Source: PLoS One. 2018 Oct 3;13(10):e0202895. doi: 10.1371/journal.pone.0202895 (PMC6169856; doi:10.1371/journal.pone.0202895)
Supplement: S1 Doc — (DOCX) [file pone.0202895.s001.docx]

**Knowledge of food taboos on Maternal Nutrition Intervention Programs**

(Phd Research project: VU University Amsterdam, Athena Institute school of Science in collaboration with Moi University Kenya, school of Arts and Social Sciences, Sociology and Psychology Department.)

**Respondent’s Code______________**

**Respondent’s Status _____________**ANC^[[1]](#footnote-1)^□ /PNC^[[2]](#footnote-2)^ □

**INTERVIEW SCHEDULE FOR ANTENATAL/POSTNATAL WOMEN SEEKING MATERNAL CARE AT A HEALTH FACILITY**

Date of Interview--------------Name of Facility---------------------------Type of Facility -----

Sub-County--------------Time Started---------Time Ended--------Estimated Length of Interview---

Interviewer’s Name----------------------------------Signature------------------------------------

**PART A: IDENTIFICATION AND INFORMED CONSENT**

***Ice Breaker Question***

Hi, I can see you have been blessed with a pregnancy, tell me how are you faring on with it?

**Informed consent and confidentiality of interviews**

Good morning/afternoon, Madam. We are a team from VU University Amsterdam and Moi University Eldoret. We are working on a project concerned with maternal nutrition and health in which you could participate. This is a PhD research study in which we would like to have an understanding on how pregnancy nutrition intervention policies are implemented in your hospital. Now, we want to know more about your knowledge, attitudes and practices on these nutritional interventions. The interview will take about 30-40 minutes. This information will be held and processed for academic purposes where by it will eventually be published in scientific journals and thesis book. All the information you provide will be kept confidential and that no information that could lead to the identification of any individual will be disclosed in any reports on the project, or to any other party. No identifiable personal data will be published. The identifiable data will not be shared with any other organisation.

The objective of this study is to get an understanding on the implementation of pregnancy nutrition intervention strategies**.** This is not to evaluate or criticize you, so please do not feel pressured to give a specific response and do not feel incompetent if you do not know the answer to a question. I am not expecting you to give a specific answer; I would like you to answer the questions honestly, telling me about what you know. Feel free to answer questions at your own pace and do not shy off from answering any question. In case you have any question regarding this research, please feel free to ask. Also, you are not obliged to answer any question you do not want to, and you may stop the interview at any time. Thank you.

At this time do you have any question to ask me about this survey before we begin? 1= Yes 2=No

*State the question and your answer below* ___________________________________________

Do I have your consent to begin the interview now? 1=Yes 2=No

Do I have your consent to have your voice recorded? 1=Yes 2=No

Consent signature of the interviewee …………………………………………………. Date ……………………………..

**Informed Consent Form for Parents/Guardians of respondents aged below 18 years**

I agree that my child/person ………………………………………(full name of child/person) for whom I am a parent/guardian may take part in the above VU University PhD research project. The project has been explained to ................………..……..(name of the child) and to me, and I have read the Participant Information Sheet, which I may keep for my records.

I understand that agreeing to take part means that I am willing to allow ………………………………………………………………(Principal Investigator) to:

- be interviewed by the researcher
- allow the interview to be audiotaped
- provide samples of blood/urine/muscle tissue/saliva/faeces __ times at __ hour/day/week intervals
- make herself available for a further interview should that be required
- allow the researchers to have access to her medical records

This information will be held and processed for academic purposes and will be eventual published in scientific journals and thesis book. I understand that any information ……………………………………. (full name of child/person) provides is confidential, and that no information that could lead to the identification of any individual will be disclosed in any reports on the project, or to any other party. No identifiable personal data will be published. The identifiable data will not be shared with any other organisation.

I also understand that ………………………’s (full name of child) participation is voluntary, that she can choose not to participate in part or all of the project, and that she or I can withdraw at any stage of the project without being penalised or disadvantaged in any way.

Participant’s Name: ..................................................................... Participant’s Age:.......................

Parent’s/Guardian’s Name ..............................................................

Signature of Parent/Guardian: ......................................................Date:................................

**PART B. Social Demographic and Health Profile of the Respondents**

1. Use the mothers’ antenatal medical cards to answer questions in this section i.e. quiz 1.1–1.17. kindly validate the answers by asking the respondents

1.1 Age ________1 2. Gravida^[[3]](#footnote-3)^ __________1 3. Parity^[[4]](#footnote-4)^ _________1 4. LMP^[[5]](#footnote-5)^ ____________ 1.5.EDD^[[6]](#footnote-6)^______ 1.6. Marital status ___________________1.7. Education ________________ 1.8.Occupation ________________1.9 Address______________1.10. HB^[[7]](#footnote-7)^ __________

1.11. Date for first ANC^[[8]](#footnote-8)^ visit _____________1.12. Maturity^[[9]](#footnote-9)^( for the first ANC visit)_________

1.13 Maturity^[[10]](#footnote-10)^ (for current date) _________1.14. The n^th^ number of the ANC visit today_______

1.15. For PNC record Birthday of the baby_______________

1.16 Previous pregnancies:

| **No.** | **Year** | **Place of birth** | **Maturity** | **Duration of labour** | **Type of delivery** | **Weight (kg)** | **Sex** | **Outcome** | **Puerperium** ^[[11]](#footnote-11)^ |
| --- | --- | --- | --- | --- | --- | --- | --- | --- | --- |
| 1 |  |  |  |  |  |  |  |  |  |
| 2 |  |  |  |  |  |  |  |  |  |
| 3 |  |  |  |  |  |  |  |  |  |
| 4 |  |  |  |  |  |  |  |  |  |
| 5 |  |  |  |  |  |  |  |  |  |

1.17 Nutritional interventions administered to the women during the ANC visit

| **No.** | **Intervention** | **Dates Given** | **Amount** |
| --- | --- | --- | --- |
| 1 | Ferrous Prescription given |  |  |
|  |  |  |  |
| 2 | Folic Acid Prescription given |  |  |
|  |  |  |  |
| 3 | IFA Prescription given |  |  |
|  |  |  |  |
| 4 | Nutritional counselling given |  |  |
|  |  |  |  |
| 5 | Macro-nutrients supplements given |  |  |
|  |  |  |  |
| 5 | De-worming tablets given to help prevent anaemia |  |  |
| 6 | IPT (Intermittent Presumptive Treatment) for malaria given |  |  |
| 6 | ITN (insecticide-treated mosquito net) issued |  |  |

2.0 Social demographic interviews with the pregnant mothers

2.1 What do you do for a living? **Probe** ___________________

2.2 What is your highest academic achievement? *Indicate the highest class accomplished*_____

2.3 What is your marital status? *Probe to get the right answer______________________*

2.4 Where do you live?

______________________ ______________ _____________________

Village/estate Sub ethnic group in a shopping centre or rural

______________________

**Response codes:** *circle the relevant code*

**Education:** ❶Pre-primary ❷ Primary incomplete ❸Primary complete ❹Secondary incomplete ❺Secondary complete ❻Higher □

**Occupation:** ❶Wage earner (company employee) ❷Casual worker ❸Self-employed (business) ❹house wife ❺Farmer ❻Permanent employee

Marital status:

| PART C: ACCESS, UTILITY AND TYPE OF ANTENATAL CARE (ANC) SERVICES | | |
| --- | --- | --- |
| Utility and Type of Antenatal Care (ANC) Services | | |
| 3.1 | I can see you started your ANC at the gestational age of *refer to* ***quiz 1.12***____ ***if at late ages ask:*** why did you not seek ANC at the health facility immediately you discovered your pregnancy? **Probe** |  |
| 3.2 | I can see you started your ANC at the gestational age of ***refer to quiz 1.12*** ____***if earlier ages*** ***(1-4 months) ask:*** what motivated you to start your ANC at the health facility this early? **Probe** |  |
| 3. | Are you normally satisfied with the nature of ANC services offered at this facility? | Yes □  No □ |
| 3.4 | ***If yes to quiz 3.3 ask*,** how? **Probe** |  |
| 3.5 | ***If no to quiz 3.3 ask***, why? **Probe** |  |
|  | Have you ever seek ANC from a traditional birth attendant? | Yes □  No □ |
|  | If yes, at what gestational age did you first seek her care? |  |
|  | ***If yes to quiz 3.4 ask****,* what form of care did you seek? **Probe** |  |
|  | ***If yes to quiz 3.4 ask*** how often do you seek her care? |  |
| 3.9 | ***If yes to quiz 3.4****,* are you usually satisfied with the ANC services offered by these traditional birth attendants? | Yes □  No □ |
| 3.10 | ***If yes to quiz 3.9 ask*** why**? Probe** |  |
| 3.11 | ***If no to quiz 3.9 ask****,* why? **Probe** |  |
| 3 | ***If has never seeked ANC services from TBA, ask:*** Are you intending to seek TBA services before you deliver **probe** |  |
| Accessibility of ANC Services | | |
| 3.12 | How far is this facility to your home? ***(in terms of minutes taken to walk) if uses other means ask in terms of transport cost.*** |  |
| 3.13 | Are you okay with this distance? | Yes □ No □ |
|  | Is this the nearest health facility that offers ANC services? | Yes □  No □ |
| 3.15 | ***If the ans. In 3.13 is no ask,*** How far is the nearest ANC facility - in terms of minutes taken to walk |  |
| 3.16 | ***If this is a further facility ask,*** why did you prefer this facility over the nearer one?  **Probe** |  |
| PART D IMPLEMENTATION FIDELITY (DOSAGE AND PARTICIPANT RESPONSIVENESS) OF THE NUTRITIONAL INTERVENTION POLICY STRATEGIES PROVIDED AT THE ANC CLINICS | | |

| Iron and Folic Acid Supplementation Strategies (show the mother the IFAS tablets and ask the following questions) | | | | |
| --- | --- | --- | --- | --- |
| 4.1 | | Have you ever come across such tablets during your current pregnancy life? | Yes □  No □ | |
| 4.2 | | ***If yes to 4.1,*** have you ever been given these tablets during your ANC visit for the current pregnancy? | Ferrous Yes □  No □  Folic acid Yes □  No □ | |
| 4.3 | | ***If yes to 4.2,*** how many times were you given? ***Probe to know the exact number issued so far*** | Number of times issued_______________  Total number of tablets issued to date: Ferrous________  Folic acid _______ | |
| 4.4 | | What dosage were you advised by the nurse to follow? ***Ask dosage for each tablet*** | ***Kindly probe and indicate correct dosage***  Ferrous _______________  Folic acid______________ | |
| 4.5 | | Did you take the dosage for the tablets as advised? ***Insist she be sincere to her response. Indicate the number of times she took them and number of days she forgot to take them, probe to get a detailed trend of taking the tablets. Count the tablets to confirm the response or ask the remaining number of tablets not yet consumed*** | **Ferrous:**  Total number of tablets consumed to date_____________  Total number of tablets not yet consumed as by now____  **folic acid:**  total number of tablets consumed to date_____________  total number of tablets not yet consumed as by now ____  **IFAs:**  Total number of tablets consumed to date_____________  Total number of tablets not yet consumed as by now____ | |
| 4.6 | | ***If dosage not correctly followed ask:***  What challenges do you face that you do not follow dosage as advised?  ***Probe to get all the possible reasons for not following the dose as recommended*** | Ferrous:  Folic acid:  IFAs | |
| 4.7 | | ***If the reasons given are related to side effects ask:*** have you ever raised the complaint to the nurse at the ANC clinic over the side effects? | Yes □  No □ | |
| 4.8 | | ***If yes,*** what advice were you given?  ***List each side effect/complain and Probe for each advice given*** | ***State each complain and list the advices given***  Complain 1:  Complain 2:  Complain 3: | |
| 4.9 | | ***If dosage is correctly followed ask:***  What motivated you to complete your dose? ***Probe*** |  | |
| 4.10 | | Other than dosage, were you given any other information regarding these tablets when they were being issued to you? **Probe on:** | Yes □  No □ | |
| 4.10 | | When you were given these tablets, were you advised on the reasons for taking them? ***Ask for each tablet*** | Ferrous: Yes □  No □  Folic acid: Yes □  No □ | |
| 4.11 | | ***For both yes and no responses, ask and probe:***  Why is it important to take ferrous/folic acid during pregnancy?  **Ask one at a time and show her the tablet** | Ferrous: ***probe***  Folic acid: ***probe*** | |
| 4.13 | | When taking these tablets, what foods are you advised to increase in your diet? ***Probe and list them*** |  | |
| 4.14 | | When taking these tablets, what foods are you advised to avoid in your diet? ***Probe and list them*** |  | |
| **General knowledge on anaemia** | | | | |
| 4.18 | | What is anaemia? |  | |
| 4.19 | | Who is vulnerable to getting anaemia? |  | |
| 4.20 | | What are the common signs and symptoms of someone who has anaemia? |  | |
| 4.21 | | What causes anaemia? |  | |
| 4.22 | | Do you think anaemia is a disease that requires treatment/drugs? **Yes □/No □**  ***Probe to indicate why it is a disease or not a disease depending on the answer*** |  | |
| 4.25 | | Do you think it is advisable for a pregnant woman to have enough iron/ blood for the entire pregnancy period? | Yes □  No □ | |
| 4.26 | | ***If yes,*** Why do you think it is important for a pregnant woman to have enough iron/blood during pregnancy? **Probe:** |  | |
| 4.23 | | Who is affected by anaemia during pregnancy? | Mother□ Baby □ Both □ | |
| 4.27 | | When a pregnant woman is anaemic: | How will it affect the mother? **Probe**  How will it affect the child in the uterus? **Probe** | |
| 4.24 | | What are the strategies of preventing anaemia during pregnancy? /how can you prevent anaemia during pregnancy? **Probe** |  | |
| 4.15 | | Can you list examples of foods you know that are rich in iron? **Probe:** |  | |
| 4.16 | | ***For each of the food listed in 4.15 ask?*** Do you eat them? | 1.  2.  3.  4.  5. | |
| 4.17 | | ***If no ask:*** why don’t you take them? | 1.  2.  3.  4. | |
| Nutritional Counselling on Need for an Adequate and Nutritious Diet Intervention Strategy | | | | |
| 5.1 | Other than anaemia related counselling, have you ever received any other nutritional counselling during your ANC visits? | | | Yes □  No □ |
| 5.2 | **If yes**, what counselling themes did you receive? **Or** what were you advised on? **Probe** | | |  |
| 5.3 | During your pregnancy, have you ever felt: | | | 1. Vomiting/ morning sickness: **Yes □/No □** 2. Constipation **Yes □/No □** 3. Heartburns **Yes □/No □** |
| 5.4 | What do you think causes vomiting, constipation, and heartburns during pregnancy? **Probe one at a time** | | | 1. Vomiting/ morning sickness 2. Constipation 3. heartburns |
| 5.3 | Have you ever been advised at the clinic on how to cope with food related complications during pregnancy?  If yes, how? ***Probe on:*** | | | 1. Vomiting/ morning sickness **Yes □/No □** 2. Constipation **Yes □/No □** 3. Heartburns **Yes □/No □** |
| 5.4 | Does a pregnant woman require special diet that is different from other members of the family?  **If yes how, probe on:** | | | Yes □/No □ |
|  | Which food is she recommended to eat in plenty? | | |  |
|  | Which foods is she recommended to avoid and why | | |  |
| 5.5 | If yes in 5.4 ask, where did you learn this information from? | | |  |
| 5.6 | Is it advisable for a pregnant woman to give birth to a big baby or a small baby? | | | **Why do you think so?** |
| 5.7 | What causes a pregnant woman to give birth to a small/underweight babies? | | |  |
| 5.8 | What health risks likely to face babies born small/underweight? **Probe** | | |  |
| 5.9 | What do you think causes neural defects/mental and physical deformities in new-born babies? **Probe** | | |  |
|  | ***If cultural beliefs not mentioned ask:*** Culturally, what do they believe causes neural defects in newborn? | | |  |
| 5.10 | Do you sleep under treated mosquito net? If no, why not? | | |  |
| 5.1 | If issued with de-wormers ask: Do you take your dose as prescribed by the nurse? | | |  |

**CONCLUSION:**

9.1 What recommendations do you have on improving the health of pregnant mothers?

9.2 Do you have any questions for us? Yes □/No □

*If yes Probe and record the question*

Thank you for your time today. We greatly appreciate you coming to talk with us today.

**INTERVIEWER ASSESSMENT**

Interviewer, please complete the questions below based on your own observation and assessment of the entire interview process and of the respondent

1. What is your assessment of the respondent's concentration and attentiveness during the interview?

1=Very good 2=Good 3=Moderate 4=Bad 5=Very bad

2. What is your evaluation of the accuracy and completeness of the respondent's answers?

1=Very high 2=High 3=Average 4=Low 5=Very low

3. What is your assessment of the respondent's comprehension of issues discussed?

1=Very good 2=Good 3=Moderate 4=Bad 5=Very bad

**INTERVIEWER NOTES**

1. Antenatal client [↑](#footnote-ref-1)
2. Postnatal client [↑](#footnote-ref-2)
3. Number of pregnancies including abortions and still births [↑](#footnote-ref-3)
4. Number of life births plus abortions [↑](#footnote-ref-4)
5. Last menstrual period [↑](#footnote-ref-5)
6. Expected date of delivery [↑](#footnote-ref-6)
7. Hemoglobin levels [↑](#footnote-ref-7)
8. Antenatal care [↑](#footnote-ref-8)
9. Gestational age (in terms of weeks) during the first antenatal care visit [↑](#footnote-ref-9)
10. Current gestational age (in terms of weeks as per today) [↑](#footnote-ref-10)
11. Postnatal complications following the mother a month after birth [↑](#footnote-ref-11)
