# Supplementary material for: “I should have started earlier, but I was not feeling ill!” Perceptions of Kalenjin women on antenatal care and its implications on initial access and differentials in patterns of antenatal care utilization in rural Uasin Gishu County Kenya
Source: PLoS One. 2018 Oct 3;13(10):e0202895. doi: 10.1371/journal.pone.0202895 (PMC6169856; doi:10.1371/journal.pone.0202895)
Supplement: S2 Doc — (PDF) [file pone.0202895.s002.pdf]

NACOSTI

NATIONAL COMMISSION FOR SCIENCE, TECHNOLOGY AND INNOVATION

Telephone: +254-20-2213471,
2241349, 310571, 2219420
Fax: +254-20-318245, 318249
Email: secretary@nacosti.go.ke
Website: www.nacosti.go.ke
When replying please quote

9th Floor, Utalii House
Uhuru Highway
P.O. Box 30623-00100
NAIROBI-KENYA

Ref: No.

Date:

2nd April, 2015

NACOSTI/P/15/2335/5353

Roselyter Monchari Rianga
Vu University Amsterdam
NETHERLANDS.

RE: RESEARCH AUTHORIZATION

Following your application for authority to carry out research on ***"Knowledge of food taboos on maternal nutrition intervention programs,"*** I am pleased to inform you that you have been authorized to undertake research in **Uasin-Gishu County** for a period ending **1st September, 2017.**

You are advised to report to **the County Commissioner and the County Director of Education, Uasin-Gishu County** before embarking on the research project.

On completion of the research, you are required to submit **two hard copies and one soft copy in pdf** of the research report/thesis to our office.

DR. S. K. LANGAT, OGW
FOR: DIRECTOR GENERAL/CEO

Copy to:

The County Commissioner
Uasin-Gishu County.

The County Directorof Education
Uasin-Gishu County.

COUNTY COMMISSIONER
UASIN GISHU COUNTY
8/4/15

THIS IS TO CERTIFY THAT:
MS. ROSELYTER MONCHARI RIANGA
of VU UNIVERSITY AMSTERDAM,
3268-1002 madaraka,has been
permitted to conduct research in
Uasin-Gishu County

on the topic: ***KNOWLEDGE OF FOOD TABOOS ON MATERNAL NUTRITION INTERVENTION PROGRAMS***

for the period ending:
1st September,2017

Permit No : NACOSTI/P/15/2335/5353
Date Of Issue : 2nd April,2015
Fee Recieved :Ksh 2,000

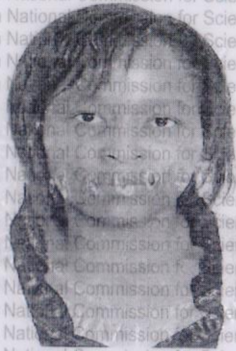

....
Applicant's Signature

....
Director General
National Commission for Science, Technology & Innovation

National Commission for Science, Technology and Innovation

CONDITIONS

1. **You must report to the County Commissioner and the County Education Officer of the area before embarking on your research. Failure to do that may lead to the cancellation of your permit**
2. **Government Officers will not be interviewed without prior appointment.**
3. **No questionnaire will be used unless it has been approved.**
4. **Excavation, filming and collection of biological specimens are subject to further permission from the relevant Government Ministries.**
5. **You are required to submit at least two(2) hard copies and one(1) soft copy of your final report.**
6. **The Government of Kenya reserves the right to modify the conditions of this permit including its cancellation without notice**

REPUBLIC OF KENYA

National Commission for Science, Technology and Innovation

RESEARCH CLEARANCE PERMIT

Serial No. A 4794

CONDITIONS: see back page

REPUBLIC OF KENYA
COUNTY GOVERNMENT OF UASIN GISHU

OFFICE OF THE COUNTY DIRECTOR – HEALTH SERVICES

TELEGRAMS "MEDICAL" ELDORET
TEL: (053) 2062711/2031421
FAX (053) 2062711

UASIN GISHU COUNTY
P O BOX 5665 – 30100
ELDORET

Date: 6th May 2015

When replying please Quote:
Ref: CDH/UG/RESEARCH/2015 (ID.NO.21832101)

Roselyter Monchari Riang'a
VU University, Amsterdam
NETHERLANDS

RE: RESEARCH AUTHORIZATION

Following an approval by the National Commission for Science, Technology and Innovation (NACOSTI) to conduct a research in Uasin Gishu County, entitled '***Knowledge of food taboos on maternal nutrition intervention programs'***, we grant you permission to carry out your research in Uasin Gishu County, for a period not exceeding 1st September 2017.

On completion of this research, you are required to submit one (1) hard copy and one (1) soft copy of the research report to my office.

By copy of this letter, I confirm to any persons concerned to accord you the necessary support; and I wish you well as you undertake the exercise.

COUNTY DIRECTOR
PREVENTIVE & PROMOTIVE SERVICES
06 MAY 2015
UASIN GISHU COUNTY
P. O. Box 5665-30100, ELDORET

Dr. Evans Kiprotich,
COUNTY DIRECTOR - P & P SERVICES
UASIN GISHU COUNTY

REPUBLIC OF KENYA

HARAMBEE

MINISTRY OF EDUCATION, SCIENCE AND TECHNOLOGY

STATE DEPARTMENT OF EDUCATION

Telegrams: **"EDUCATION",** Eldoret
Telephone: **053-2063342** or **2031421/2**
Mobile : **0719 12 72 12/0732 260 280**
Email: cdeuasingishucounty@yahoo.com
: cdeuasingishucounty@gmail.com

Office of The County Director of Education,
Uasin Gishu County,
P.O. Box 9843-30100,
ELDORET.

When replying please quote:

Ref: No. MOEST/UGC/TRN/9/VOL II/11

8TH APRIL, 2015

Roselyter Monchari Rianga
Vu University Amsterdam
Netherlands

RE: RESEARCH AUTHORIZATION

This office has received your letter requesting for authority to allow you carry out research on **"Knowledge of Food Taboos on Maternal Nutrition Intervation Programs",** Within Uasin Gishu County."

We wish to inform you that the request has been granted for a period ending **1st September,2017.** The authorities concerned are therefore requested to give you maximum support.

We take this opportunity to wish you well during this research.

KIPLAGAT J. ROP
For: **COUNTY DIRECTOR OF EDUCATION**
UASIN GISHU COUNTY

COUNTY DIRECTOR OF EDUCATION
08 APR 2015
P.O. Box 9843-30100, ELDORET

ISO 9001
BUREAU VERITAS
Certification
UKAS

VU VRIJE UNIVERSITEIT AMSTERDAM | International Office

CIS Centre for International Cooperation (CIS-VU)
POSTAL & VISITING ADDRESS: De Boelelaan 1105, 1081 HV Amsterdam, The Netherlands
Web: www.cis.vu.nl

MOI UNIVERSITY
Moi University
P.O. Box 3900 – 03100
Eldoret

Amsterdam, 25 February 2015

To whom it may concern

On behalf of the VU University Amsterdam, I am pleased to introduce to you Ms Roselyter M. Riangá. Ms Riangá, also staff member of Moi University in Kenya, is currently enrolled in the PhD programme at the Athena Institute of the VU University Amsterdam in the Netherlands. One of her major tasks is to conduct research on *Knowledge of Food Taboos on Maternal Nutrition Intervention Programs*. This research is funded by a project titled *A Sustainable Approach to Livelihood Improvement* (ASALI), which is a joint undertaking of Moi University, South Eastern Kenya University and VU University Amsterdam. The major part of Ms Riangá's research will be conducted in Kenya, particularly in the Uasin Gishu County. To do so, Ms Riangá will spend at least two periods of 3 to 6 months (during 2015-2017) in Kenya to gather data within local communities and visit relevant institutes. The remainder time serves for data analysis and thesis writing at the VU University in the Netherlands.

The main supervisor of Ms Riangá in the Netherlands is Prof. Dr Jaqueline Broerse of the Athena Institute. The local supervisor is Prof Dr Anne Nangulu of Moi University.

We kindly ask the support of your good office to assist Ms Riangá with her requests necessary for successful completion of her Ph.D research.

Thank you so much in advance for your collaboration and support.

Yours sincerely,

D. Snelder

Dr Denyse J Snelder
ASALI Project Coordinator
CIS -VU International Office
VU University Amsterdam
De Boelelaan 1107
1081 HV Amsterdam
The Netherlands

Moi University
P.O. Box 3900 – 03100
Eldoret

MOI UNIVERSITY
SCHOOL OF ARTS AND SOCIAL SCIENCES
DEPARTMENT OF SOCIOLOGY AND PSYCHOLOGY

Tel: 254-053-43620
P.O. Box 3900 - 30100
ELDORET - KENYA

FAX: 254-053-43047
Telex: 35047 MOIVARSITY
E-Mail:vcmu@irmmoi.com

6th March 2015

The Chief Executive Officer/Secretary
National Commission for Science Technology and Innovation
P.O. Box 30623-00100,
Nairobi, Kenya

Dear Sir,

RE: LETTER OF AFFILIATION FOR Ms ROSELYTER M RIANG'A

This is to certify that the above named is a member of staff in the Department of Sociology and Psychology in this University. Ms. Rianga is currently enrolled for a doctorate degree at VU University of Amsterdam in the Netherlands and is due to proceed for field work/data collection. Her research proposal is titled: ''KNOWLEDGE OF FOOD TABOOS ON MATERNAL NUTRITION INTERVENTION PROGRAMMES. The study will be conducted in Uasin Gishu County. The research will be in two phases with the first phase taking place between March 2015 to October 2015. The second phase will be in 2016 – 2017.

Any assistance accorded to her for procurement of the research permit will be appreciated.

Yours Sincerely

DR JAMIN R M MASINDE (PhD)
E-Mail: drjaminmasinde@yahoo.co.uk
HEAD OF DEPARTMENT, SOCIOLOGY AND PSYCHOLOGY
MOI UNIVERSITY

DECLARATION

DECLARATION BY CANDIDATE

This proposal is my original work and has not been presented for a degree award in any university. No part of this proposal may be produced without prior permission from the author and/or VU University Amsterdam.

25-02-2015

RIANG'A ROSELYTER **DATE**

DECLARATION BY SUPERVISORS

This proposal has been submitted with our approval as university supervisors.

26-02-2015

Prof. Dr. Jacqueline.E.W. Broerse **DATE**

Athena Institute

VU University Amsterdam

Faculty of Earth and Life Sciences

Prof. Anne Nangulu

6/3/2015

Prof. Ann Nangulu **DATE**

Department of History

Moi University

School of Arts and Social Studies

KCB

Date: 10/03/2015

CREDIT ADVICE
CASH DEPOSIT

KCB THIKA

Account AT KCB KIPANDE HOUSE

ACCOUNT DETAILS

A/C NO: 1104162547
A/C REF: 005241970364
NAT COMM FOR SCI ,TECH AND INNOV
Current Account-Corp Inst. Bankin

KENYA COMMERCIAL BANK LTD. THIKA 10 MAR 2015 TELLER NO. 24

We have credited your above account with 2,000.00 KES

Kenya Shillings TWO THOUSAND ONLY

CASH PAID IN BY: ROSELYTER M RIANGA
RESEARCH PERMIT

Signature :.........................

Transaction Number: TT150697PWZZ at 11:45:39 On 10/03/2015

Thank you for banking with us. You were served by: OLIVE NUNGARI KIBURU

*** Advice not valid unless Transaction Number is shown ***

PAP.1064 Beam Version 11 (Revised Sep 2010)
